# Pediatric foot anthropometry and its correlation with growth assessment

**DOI:** 10.1038/s41598-026-43428-5

**Published:** 2026-03-13

**Authors:** Willy Barinem Vidona, Titilayo Opeyemi Bolaji, Collins Nduka Esomchi

**Affiliations:** 1 Department of Anatomy, Faculty of Basic Medical Sciences, Edo State University Uzairue, Uzairue, Edo State Nigeria; 2 Department of Human Anatomy, Faculty of Basic Medical Sciences, State University of Medical and Applied Sciences, Igbo-Eno, Enugu State, Nigeria

**Keywords:** Pediatric foot anthropometry, Foot length, Foot width, Age, Height, Growth, Nigeria, Children, Anatomy, Health care, Health occupations, Medical research, Risk factors

## Abstract

Foot dimensions, particularly length and width, are essential anthropometric parameters often used in health, ergonomics, and footwear design. This study aimed to investigate the correlation between foot dimensions, age, and height among children aged 4 to 12 years in Ekpoma, Edo State, Nigeria. A cross-sectional descriptive survey was conducted with 389 children, randomly selected from schools and community centres. Data were collected using standardised anthropometric measurements, including foot length, foot width, and height, ensuring accuracy and consistency. Pearson’s correlation and independent t-tests were used to examine relationships among variables and to identify sex-based differences. Descriptive statistics revealed variations in foot dimensions across the age groups, with a mean foot length of 19.49 cm and a mean foot width of 6.87 cm. Foot length showed moderate-to-strong correlations with age in younger children (overall *r* = 0.549, *p* < 0.001) and a strong correlation with height (*r* = 0.652, *p* < 0.001), while foot width exhibited weaker positive correlations with age (r range 0.254–0.513) and height (*r* = 0.233, *p* < 0.001). No significant sex differences were observed (all *p* > 0.05). The findings highlight the progressive changes in foot dimensions with age and height and their potential applications in pediatric health, footwear design, and ergonomic planning.

## Introduction

Anthropometric measurements, which involve the systematic assessment of the human body, are pivotal in understanding growth and development across various populations^1^. Among these measurements, foot dimensions have emerged as a key area of interest due to their potential role as indicators of overall body growth and development in children^2^. Investigating the relationship between foot dimensions and age is essential for several reasons, including monitoring growth patterns, assessing developmental stages, and identifying potential health issues^3^.

Foot dimensions (length, width, and arch height) change markedly during childhood as part of broader physical growth, shaped by genetic, nutritional, and environmental factors^4^. Feet often show rapid growth that precedes or aligns with milestones like puberty, serving as an early indicator of developmental timing^5–7^. Vandervael’s model indicates that lower limb maturation peaks pre-puberty, with foot ossification preceding that of long bones^8,9^. Cross-sectional studies confirm accelerated foot growth just before pubertal onset (around 7–9 years in girls, 8–10 in boys), often preceding peak height velocity by 1–2 years and linking to Tanner stages^4,5,7,10,11^. Variations or deviations from expected patterns may signal endocrine, nutritional, or developmental issues, supporting early detection and intervention^4,9^.

Moreover, anthropometric measurements of foot dimensions can help identify potential deviations from expected growth trajectories^4^. For example, unusually rapid or slow growth in foot size may indicate underlying health issues such as endocrine disorders or nutritional deficiencies^12^. In developmental disorders like Down syndrome, boys exhibit significantly shorter, narrower, and flatter feet (longitudinally and transversely) compared to typically developing peers^13^, highlighting foot anthropometry as a marker of atypical growth and development. Early identification of such deviations enables timely intervention and management, which can significantly improve overall health outcomes^9^.

A significant aspect of studying foot dimensions in children is their correlation with age and height. As children age, their feet undergo changes in size and shape. Recent Nigerian data have confirmed a strong positive correlation between foot length and height in school-aged children, supporting its utility as a reliable proxy for stature estimation in local populations^14^. This correlation provides a framework for establishing growth patterns and evaluating whether a child’s development is proceeding within the expected range^15^. Longitudinal studies that track foot growth over time can reveal how quickly feet grow relative to other body parts and how these patterns align with developmental stages^3^.

This study was conducted to investigate the anthropometric foot dimensions and its correlation with age and height in children and to identify any significant differences in between males and females in the sample group.

Therefore, this cross-sectional study aimed to address the following specific research questions:

1. What is the strength and direction of the correlation between foot dimensions (length and width) and age in Nigerian children aged 4–12 years?
2. What is the strength and direction of the correlation between foot dimensions (length and width) and height in this population?
3. Are there significant sex differences in foot dimensions, age, or height among these children?

We hypothesised that:

- Foot length and width would show significant positive correlations with both age (moderate to strong for length) and height (strong for length), consistent with progressive skeletal growth during childhood.
- No statistically significant sex differences would be observed in foot dimensions or height in this prepubertal age group, as hormonal influences on sexual dimorphism typically emerge later during adolescence.

## Methods

This cross-sectional descriptive study was conducted in accordance with the STROBE guidelines for reporting observational studies.

### Study design

A cross-sectional survey assessed correlations between foot anthropometric dimensions (length and width), age, and height in children aged 4–12 years.

### Setting

The study was conducted in Ekpoma, Esan West Local Government Area, Edo State, Nigeria (latitude 6°45’N, longitude 6°5’–6°8’E), from January to June 2025. Participants were recruited from selected primary schools and community centres serving a diverse population (students, traders, artisans, civil servants), with Ambrose Alli University contributing many participants.

### Participants

Children aged 4–12 years attending the selected schools/centres were eligible if apparently healthy.

Inclusion Criteria.

- Age 4–12 years (verified by birth certificate, school records, or parental report in completed years).
- Both males and females.
- No self-reported or observed acute illness at recruitment.
- Written informed consent from parents/guardians and verbal assent from the child (where age-appropriate, e.g., ≥ 7 years).

Exclusion Criteria.

- Diagnosed congenital anomalies, foot deformities, or growth-affecting conditions (e.g., endocrine disorders, chronic malnutrition, skeletal dysplasias).
- Severe physical disabilities or mobility impairments affecting measurements.
- Acute foot injuries, infections, or swelling at measurement time.
- Recent lower limb surgery or trauma.
- Refusal of consent/assent or withdrawal during the process.
- Incomplete data.

**Fig. 1:**
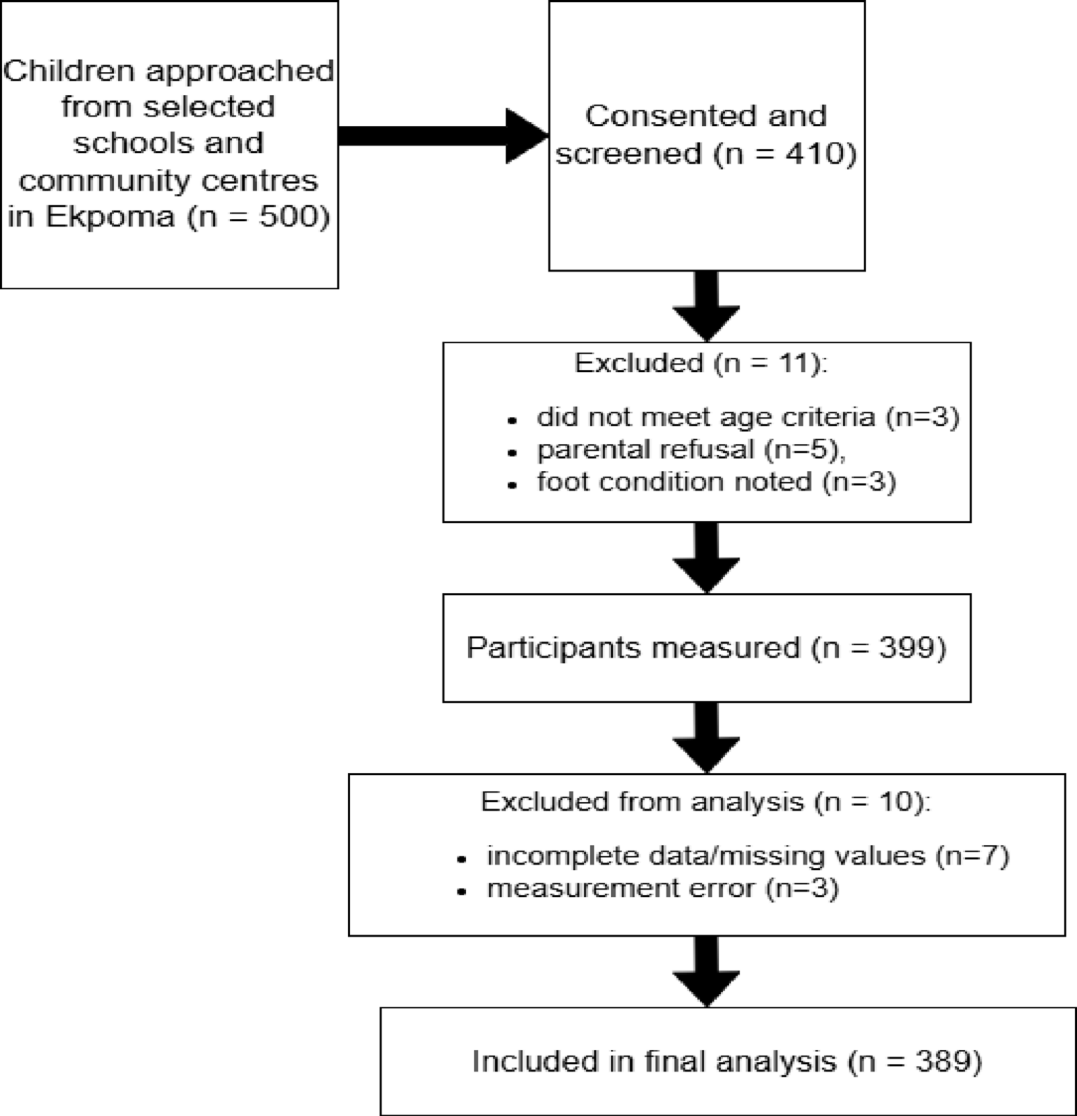
Flow diagram of participant recruitment and inclusion in the study. (adapted from STROBE guidelines).

A total of 500 children were approached via stratified random sampling (strata by age group [4–6, 7–9, 10–12 years] and sex); 389 were included in the final analysis after exclusions (see Fig. 1 for participant flow).

### Recruitment procedure

Permission was obtained from school authorities and community centre leaders via formal letters detailing study objectives, procedures, risks/benefits, and voluntary participation. Ethical approval was granted by the Ambrose Alli University Ethics Committee (number 026/25).

Eligible classes/age groups were identified with teacher assistance, and potential participants were stratified by age and sex. Parents/guardians attended information sessions (e.g., PTA meetings or community gatherings) for verbal/written explanations (in English and local languages as needed) and consent form distribution. Written consent was obtained; no incentives were offered. Consenting children were scheduled for measurement sessions at the school/centre to minimise disruption.

### Data collection procedure

Measurements were performed by trained research assistants (anthropometry-experienced from the Department of Anatomy) under the corresponding author’s supervision, following standardised protocols to minimise inter-observer error.

### Training and quality control

Assistants completed a 2-day workshop with theoretical and practical pilot testing (~ 20 non-study children), achieving inter-rater reliability (intraclass correlation > 0.95 for all measures). Instruments were calibrated daily/weekly; one supervisor spot-checked ~ 10% of measurements. Data were recorded immediately on standardised forms and double-entered into a secure database.

### Setting and conditions

Measurements occurred in a quiet, well-lit room at ambient temperature (22–28 °C) during morning hours (8–11 AM) to reduce diurnal variation. Participants were barefoot, in light clothing, after a brief rest (5 min); they stood naturally on a flat surface with weight evenly distributed.

### Measurement instruments

- Height: Portable stadiometer (Seca 213; Seca GmbH, Germany; accuracy ± 0.1 cm; range 20–205 cm), calibrated weekly against a standard rod.
- Body weight: Calibrated digital scale (Seca; accuracy ± 0.1 kg).
- Foot dimensions: Flexible anthropometric tape/ruler (Lufkin; accuracy ± 0.1 cm), checked daily against a fixed metal ruler for wear/stretch.
- All instruments were cleaned between participants.

### Detailed measurement steps

1. Demographics: Age (completed years from records/parental report) and sex recorded.
2. Height: Participant stood erect (heels together, buttocks/shoulders/occiput against vertical board, knees straight, head in Frankfurt plane). Headboard lowered to compress hair; read at eye level to nearest 0.1 cm. Two measurements; average used (third if difference > 0.5 cm).
3. Weight: Measured to nearest 0.1 kg; BMI calculated as weight (kg) / height² (m).
4. Foot dimensions (right foot preferred):

- Length: Ruler along medial border from posterior heel to longest toe tip.
- Width: Widest transverse distance at the metatarsal heads level.

Two measurements per dimension; average to nearest 0.1 cm (third if discrepancy > 0.2 cm, median used).

### Bias

Measurement bias was minimised through standardised protocols, training, calibrated instruments, repeated/averaged measures, and supervisor checks. Selection bias was reduced via stratified random sampling within sites; no incentives influenced participation.

### Study size

Estimated for *r* ≈ 0.6 (foot length-height) using correlation power formula (α = 0.05, power = 80%): *n* ≈ 350–400; targeted 400 to account for attrition.

### Quantitative variables and statistical methods

All analyses were conducted using SPSS version 25 (IBM Corp., Armonk, NY, USA). Continuous variables were summarised using mean ± standard deviation (SD), median (interquartile range, IQR), and minimum–maximum values. Normality was assessed with the Shapiro–Wilk test; most variables showed approximate normality, supporting the use of parametric tests. Pearson correlation coefficients were calculated to examine relationships between foot dimensions (length and width) and age/height, with strength interpreted using Cohen’s conventions (> 0.50 strong, 0.30–0.50 moderate, < 0.30 weak). Independent samples t-tests compared means between sexes. Statistical significance was set at *p* < 0.05 (two-tailed). No adjustment for multiple comparisons was applied, given the exploratory nature of the study. Complete-case analysis was used, with no imputation for missing data. Age-category subgroup analyses were considered secondary/exploratory.

## Results

### Descriptive statistics

A total of 389 children (mean age 8.11 ± 2.52 years) were included. Table 1 summarises the descriptive characteristics of the study population.

**Table 1:** Descriptive Characteristics of the Study Population (*n* = 389).

Variable	Mean ± SD	Median (IQR)	Min – Max
Age (years)	8.11 ± 2.52	8.0 (6.0–10.0)	4–12
Height (cm)	130.2 ± 15.4	129.5 (118.0–142.0)	95.0–165.0
Weight (kg)	28.5 ± 8.2	27.0 (22.5–33.5)	15.0–55.0
BMI (kg/m²)	16.8 ± 2.1	16.6 (15.3–18.1)	12.5–24.0
Foot length (cm)	19.49 ± 2.55	19.4 (17.5–21.3)	14.0–25.0
Foot width (cm)	6.87 ± 0.76	6.9 (6.3–7.4)	5.0–9.0

### Correlations with age

Foot dimensions showed positive associations with age in most groups (Tables 2 and 3). Correlation strength was interpreted using Cohen’s conventions (> 0.5 strong, 0.3–0.5 moderate, < 0.3 weak).

**Table 2:** Correlation between foot dimensions and age by age group.

Age (years)	Foot Dimension	*r*	*p*-value	Inference	Strength
4	Length	0.413	0.015	S	Moderate
4	Width	0.108	0.544	NS	Weak
5	Length	0.245	0.093	NS	Weak
5	Width	0.281	0.053	NS	Weak
6	Length	0.715**	< 0.001	S	Strong
6	Width	-0.191	0.263	NS	Weak
7	Length	0.626**	< 0.001	S	Strong
7	Width	0.391**	0.006	S	Moderate
8	Length	0.535**	0.001	S	Strong
8	Width	0.088	0.598	NS	Weak
9	Length	0.342*	0.017	S	Moderate
9	Width	0.483**	0.001	S	Moderate
10	Length	0.053	0.702	NS	Weak
10	Width	0.201	0.144	NS	Weak
11	Length	0.674**	< 0.001	S	Strong
11	Width	0.505**	0.001	S	Strong
12	Length	0.530**	< 0.001	S	Strong
12	Width	0.390*	0.013	S	Moderate

*Correlation is significant at the 0.05 level (2-tailed).

**Correlation is significant at the 0.01 level (2-tailed).

S = Significant; NS = Not Significant.

**Table 3:** Correlation between foot dimensions and age by age category.

Age Category	Foot Dimension	*r*	*p*-value	Inference	Strength
4–6	Length	0.583	< 0.001	S	Strong
4–6	Width	0.254	0.005	S	Weak
7–9	Length	0.549	< 0.001	S	Strong
7–9	Width	0.513	< 0.001	S	Strong
10–12	Length	0.036	0.678	NS	Weak
10–12	Width	0.422	< 0.001	S	Moderate

*Correlation is significant at the 0.05 level (2-tailed).

**Correlation is significant at the 0.01 level (2-tailed).

S = Significant; NS = Not Significant.

Foot length exhibited moderate-to-strong correlations with age in younger groups (4–9 years), but the relationship weakened markedly or became non-significant in older children (10–12 years), suggesting a plateau in linear foot growth. Foot width showed more persistent (though generally weaker-to-moderate) associations across ages.

### Correlations with height

Both foot dimensions correlated positively with height (Table 4), with foot length showing a strong association (*r* = 0.652, *p* < 0.001) and foot width a weak-to-moderate one (*r* = 0.233, *p* < 0.001).

**Table 4:** Correlation between foot dimensions and height (*n* = 389).

Foot Dimension	*r*	*p*-value	Inference	Strength
Length	0.652	< 0.001	S	Strong
Width	0.233	< 0.001	S	Weak-to-moderate

### Sex differences

Sex-specific analyses revealed no statistically significant differences between males and females in age, height, foot length, or foot width (all *p* > 0.05; Table 5). Consequently, foot dimension data were pooled for primary correlation analyses to increase statistical power and simplify presentation. Exploratory sex-stratified correlations produced similar patterns and strengths.

**Table 5:** Sex differences in age, height, foot length, and foot width.

Foot Dimension	Male	Female	T-test	*P*-value	Inference
Age (Years)	8.17 ± 2.53	8.06 ± 2.51	0.418	0.676	NS
Height (cm)	130.5 ± 15.6	129.9 ± 15.2	0.138	0.890	NS
Length (cm)	19.44 ± 2.36	19.54 ± 2.73	-0.374	0.709	NS
Width (cm)	6.67 ± 1.11	6.90 ± 0.41	-0.213	0.832	NS

## Discussion

This cross-sectional study of Nigerian children aged 4–12 years in Ekpoma revealed distinct foot growth patterns. Foot length exhibited moderate-to-strong positive associations with age during early and middle childhood (*r* = 0.342–0.715 in 4–9-year groups), consistent with rapid longitudinal skeletal elongation typical of this developmental phase. Notably, this relationship weakened markedly or became non-significant in the 10–12-year group (*r* = 0.036), suggesting an earlier plateau in foot length growth compared to overall height velocity, in line with reports that peak foot length often precedes the adolescent spurt^10,16,17^. Recent large-scale surveys have further demonstrated that foot length correlates strongly with height and serves as an early indicator of pubertal development, often preceding peak height velocity^11^. Similar patterns of stronger foot length-height correlations and age-related stabilization have been reported in European schoolchildren, where foot growth often precedes Tanner stage II changes^10^. Foot width, by contrast, showed more sustained associations across the age range (weak-to-moderate overall, with *r* = 0.422 remaining significant in older children), likely reflecting continued transverse adaptation to increasing body mass, postural demands, and physical activity^18^. Recent studies indicate that midfoot width and arch parameters continue to evolve with age and BMI, supporting the observed persistence of width correlations even as length stabilizes^19^.

Foot length demonstrated a particularly strong correlation with height (*r* = 0.652), reinforcing its established value as a practical, non-invasive proxy for overall stature in pediatric populations^20^. A similar strong correlation has been reported in a large cohort of 1677 Nigerian schoolchildren^14^. The weaker but significant association for foot width (*r* = 0.233) aligns with biomechanical principles of transverse expansion to maintain stability and load distribution as body size increases^21,22^.

No meaningful sex differences were observed in age, height, or foot dimensions (all *p* > 0.05), consistent with evidence that sexual dimorphism in foot size remains minimal before puberty due to limited gonadal hormone influence^23,24^. Emerging evidence suggests that sex-based differences in foot morphology (e.g., wider forefoot in boys) may begin to appear around Tanner stage II or later, consistent with our findings of no significant differences in this prepubertal sample^25^. Exploratory sex-stratified correlations yielded comparable patterns in both groups, justifying pooled analyses and supporting generalizability within this prepubertal age range.

These findings carry practical implications for pediatric health surveillance and footwear provision, especially in low-resource settings. Foot measurements provide an accessible marker for monitoring developmental timing and detecting early deviations potentially linked to nutritional or endocrine factors^4^. The differential timing of length versus width changes is particularly relevant for footwear design during active growth phases^2^. Excessive functional room has been shown to adversely affect foot morphology; for example, in 7-year-old children, greater length excess was associated with flatter longitudinal arches (reduced Clarke’s angle) and, in boys, increased hallux valgus angles, potentially predisposing to long-term structural issues^26^. Inadequate width allowance may restrict natural expansion, while overly generous fit can cause instability, compensatory toe gripping, or abnormal muscle activation^27,28^. Maintaining recommended allowances (typically 8–12 mm length excess and sufficient width) supports healthy arch development, prevents deformities, and promotes functional foot growth^18,26^. Regional studies in diverse populations have similarly highlighted the importance of age-appropriate footwear to prevent deformities during rapid growth phases^29^. In Nigerian settings, where access to properly fitted, affordable footwear remains limited, these insights could inform culturally and economically appropriate guidelines.

### Study limitations

The cross-sectional design precludes causal inferences and individual trajectory analysis; longitudinal studies would provide stronger evidence of dynamic changes. Reliance on flexible rulers, while practical in field settings, may introduce minor variability compared with specialised instruments (e.g., podoscopes or digital callipers). Although weight and BMI were recorded, their potential moderating effects on foot growth were not formally explored. The sample, drawn from schools and community centres in one urban-rural area of Edo State, may not fully represent Nigeria’s ethnic and geographic diversity. Arch height, plantar pressure, or other postural parameters were not assessed, limiting insights into foot function and posture. Finally, while stratified random sampling was used, consent and school-based recruitment may have introduced some selection bias.

In summary, this study provides novel, region-specific data on foot anthropometry and its correlations with age and height in an underrepresented Nigerian pediatric population. By documenting stronger length associations in younger ages, more persistent width adaptation, absence of prepubertal sex differences, and practical implications for growth monitoring and footwear, it addresses a clear gap in sub-Saharan African evidence. These findings offer a foundation for context-appropriate health screening tools, culturally sensitive footwear recommendations, and future research on environmental and nutritional influences on foot development in African children. This ultimately supports improved pediatric care and ergonomic interventions in resource-constrained settings.

## Conclusion

In Nigerian children aged 4–12 years, foot length exhibited moderate-to-strong correlations with age in younger groups (weakening later) and a strong correlation with height, while foot width showed persistent weaker-to-moderate associations with both. No sex differences were found in foot dimensions, age, or height, aligning with expectations in prepuberty.

These results support the hypotheses that foot anthropometry reflects overall growth patterns and that sexual dimorphism remains minimal before puberty. The findings provide novel, context-specific evidence from an underrepresented population, offering practical value for pediatric growth monitoring and footwear/orthotic design in low-resource African settings.

### Recommendations

Future studies should adopt longitudinal designs to track individual growth trajectories and better understand the dynamic relationship between foot dimensions, age, and height. Inclusion of additional parameters (e.g., arch height, plantar pressure, nutritional status) and broader geographic sampling in Nigeria would enhance generalisability. Such designs could also incorporate local economic and cultural factors to improve adherence.

## Data Availability

The datasets generated and analysed during the current study are available from the corresponding author upon reasonable request.

## References

[1] Ezemagu, U. K. et al. Fetal Sex and Maternal Stature vs. Spontaneous Vaginal Birth: Considering Sex Specific State-of-the-Art Anthropometric Investigations at Term Pregnancy. *J. Obstet. Gynecol. Cancer Res.* **9**, 174–184. 10.30699/jogcr.9.2.174 (2024).

[2] Squibb, M., Sheerin, K. & Francis, P. Measurement of the Developing Foot in Shod and Barefoot Paediatric Populations: A Narrative Review. *Child. (Basel Switzerland)*. **9**, 750. 10.3390/children9050750 (2022). 10.3390/children9050750 PMC9139892 35626927

[3] Drefus, L. C., Kedem, P., Mangan, S. M., Scher, D. M. & Hillstrom, H. J. Reliability of the Arch Height Index as a Measure of Foot Structure in Children. *Pediatr. Phys. Ther. Off Publ Sect. Pediatr. Am. Phys. Ther. Assoc.* **29**, 83–88. 10.1097/PEP.0000000000000337 (2017). 10.1097/PEP.0000000000000337 27984478

[4] Egwu, O. A., Esom, E. A., Finbarrs-Bello, E. & Ekechukwu, S. Morphometric Study of the Navicular Bone in a Nigerian Population: A Direct Measurement Study. *J. Am. Podiatr. Med. Assoc.* **112**. 10.7547/20-254 (2022).

[5] Escalona-Marfil, C. et al. Valores normativos para determinar un pie plano o cavo. *Rev. Española Podol*. **30**, 15–23. 10.20986/revesppod.2019.1540/2019 (2019).

[6] Banwell, H. A., Paris, M. E., Mackintosh, S. & Williams, C. M. Paediatric flexible flat foot: how are we measuring it and are we getting it right? A systematic review. *J. Foot Ankle Res.* **11**, 21. 10.1186/s13047-018-0264-3 (2018). 29854006 10.1186/s13047-018-0264-3 PMC5975578

[7] de Carvalho, B. K. G. et al. The influence of gender and body mass index on the FPI-6 evaluated foot posture of 10- to 14-year-old school children in São Paulo, Brazil: a cross-sectional study. *J. Foot Ankle Res.* **10**, 1. 10.1186/s13047-016-0183-0 (2017). 28670344 10.1186/s13047-016-0183-0 PMC5488407

[8] Mueller, S., Carlsohn, A., Mueller, J., Baur, H. & Mayer, F. Influence of Obesity on Foot Loading Characteristics in Gait for Children Aged 1 to 12 Years. *PLoS One*. **11**, e0149924. 10.1371/journal.pone.0149924 (2016). 26914211 10.1371/journal.pone.0149924 PMC4767217

[9] Jankowicz-Szymańska, A., Wódka, K., Kołpa, M. & Mikołajczyk, E. Foot longitudinal arches in obese, overweight and normal weight females who differ in age. *Homo* **69**, 37–42. 10.1016/j.jchb.2018.03.001 (2018). 29709300 10.1016/j.jchb.2018.03.001

[10] González-Elena, M. L., Fernández-Espejo, E., Castro-Méndez, A., Guerra-Martín, M. D. & Córdoba-Fernández, A. A Cross-Sectional Study of Foot Growth and Its Correlation with Anthropometric Parameters in a Representative Cohort of Schoolchildren from Southern Spain. *Int. J. Environ. Res. Public. Health*. **18**, 4031. 10.3390/ijerph18084031 (2021). 33921266 10.3390/ijerph18084031 PMC8068955

[11] Wu, H-H. et al. Association of height, foot length, and pubertal development in children aged 3–18: a cross-sectional survey in China. *Front. Public. Heal*. **12**, 1322333. 10.3389/fpubh.2024.1322333 (2024). 10.3389/fpubh.2024.1322333 PMC10894912 38410665

[12] Boswell, S. B. et al. Musculoskeletal manifestations of endocrine disorders. *Clin. Imaging*. **38**, 384–396. 10.1016/j.clinimag.2014.02.014 (2014). 24642251 10.1016/j.clinimag.2014.02.014

[13] Puszczałowska-Lizis, E. et al. Foot Structure in Boys with Down Syndrome. *Biomed. Res. Int.* **2017**, 7047468. 10.1155/2017/7047468 (2017). 28904967 10.1155/2017/7047468 PMC5585551

[14] Bafor, A., Chibuzom, C. N. & Mbanuzuru, A. V. Correlation Between Foot Length and Height in a Cohort of 1677 Nigerian School Children. *J. Niger Acad. Med.* **2**, 60–64. 10.4103/jnam.jnam_43_22 (2023).

[15] Sadeghi-Demneh, E. et al. Flatfoot and obesity in school-age children: a cross-sectional study. *Clin. Obes.* **6**, 42–50. 10.1111/cob.12125 (2016). 26639935 10.1111/cob.12125

[16] Busscher, I. et al. The growth of different body length dimensions is not predictive for the peak growth velocity of sitting height in the individual child. *Eur. Spine J. Off Publ Eur. Spine Soc. Eur. Spinal Deform Soc. Eur. Sect. Cerv. Spine Res. Soc.* **20**, 791–797. 10.1007/s00586-010-1584-6 (2011). 10.1007/s00586-010-1584-6 PMC3082670 20936309

[17] Reeves, J., Buckley, R. & Dixon, S. Differences in Foot Morphology across Age Groups for Women Active in Sport. *Gerontology* **70**, 1267–1283. 10.1159/000541732 (2024). 39369693 10.1159/000541732 PMC11633873

[18] Puszczałowska-Lizis, E., Lizis, S. & Mikuláková, W. Variability of foot growth in width in relation to length among 3-year-old girls and boys. *Acta Bioeng. Biomech.* **25**, 79–84 (2023). 38314537

[19] Escalona-Marfil, C. et al. Children’s foot parameters and basic anthropometry — do arch height and midfoot width change? *Eur. J. Pediatr.* **182**, 777–784. 10.1007/s00431-022-04715-1 (2023). 36478295 10.1007/s00431-022-04715-1 PMC9899181

[20] Masanovic, B., Gardasevic, J. & Arifi, F. Relationship between foot length measurements and body height: a prospective regional study among adolescents in northern region of Kosovo. *Anthropol. (Czech Republic)*. **57**, 227–233 (2019).

[21] Chow, T-H., Chen, Y-S., Chou, H-T., Chang, C-C. & Lin, M-H. Biomechanical Adaptations in Foot Characteristics Among Elite Male Weightlifters: A Cross-Sectional Comparative Study. *Med. Sci. Monit. Int. Med. J. Exp. Clin. Res.* **31**, e950416. 10.12659/MSM.950416 (2025). 10.12659/MSM.950416 PMC12574155 41139217

[22] Kozel, M. et al. Biomechanical and Posturographic Aspects of the Foot as a Basis of the Sport’s Postural Characteristics. *Appl. Sci.* **16**, 434. 10.3390/app16010434 (2025).

[23] Fessler, D. M. T., Haley, K. J. & Lal, R. D. Sexual dimorphism in foot length proportionate to stature. *Ann. Hum. Biol.* **32**, 44–59. 10.1080/03014460400027581 (2005). 15788354 10.1080/03014460400027581

[24] Etchell, A. et al. A systematic literature review of sex differences in childhood language and brain development. *Neuropsychologia* **114**, 19–31. 10.1016/j.neuropsychologia.2018.04.011 (2018). 29654881 10.1016/j.neuropsychologia.2018.04.011 PMC5988993

[25] Domínguez, M. P. et al. Anthropometric Foot Variations in Children: A Cross-Sectional Study Supporting Sex-Based Last Design. *J. Foot Ankle Res.* **18**, e70069. 10.1002/jfa2.70069 (2025). 40746028 10.1002/jfa2.70069 PMC12314192

[26] Puszczalowska-Lizis, E., Lukasiewicz, A., Lizis, S. & Omorczyk, J. The impact of functional excess of footwear on the foot shape of 7-year-old girls and boys. *PeerJ* **9**, e11277. 10.7717/peerj.11277 (2021). 33976980 10.7717/peerj.11277 PMC8063877

[27] Buldt, A. K. & Menz, H. B. Incorrectly fitted footwear, foot pain and foot disorders: a systematic search and narrative review of the literature. *J. Foot Ankle Res.* **11**, 43. 10.1186/s13047-018-0284-z (2018). 30065787 10.1186/s13047-018-0284-z PMC6064070

[28] Jeong, D-H., Jeong, H-M., Park, D-J., Sung, J-Y. & Lee, K-L. Effects of Toe-Strengthening Exercises on Medial Longitudinal Arch Height, Muscle Stiffness, and Functional Movement. *Appl. Sci.* **14**, 9842. 10.3390/app14219842 (2024).

[29] Martín-Casado, L., Palomo-Fernández, I., Aldana-Caballero, A., Baltasar-Fernandez, I. & Marcos-Tejedor, F. High Rate of Change of the Foot in Ecuadorian Children: The Need for Proper Shoe Design. *Child. (Basel Switzerland)*. **11**. 10.3390/children11060749 (2024). 10.3390/children11060749 PMC11201716 38929328

